# Spectroscopic imaging with single acquisition ptychography and a hyperspectral detector

**DOI:** 10.1038/s41598-019-48642-y

**Published:** 2019-08-22

**Authors:** Darren J. Batey, Silvia Cipiccia, Frederic Van Assche, Sander Vanheule, Juriaan Vanmechelen, Matthieu N. Boone, Christoph Rau

**Affiliations:** 1 Diamond Light Source, Harwell Science and Innovation Campus, Fermi Avenue, Didcot, OX11 0QX UK; 2 UGCT-RP, Dept. Physics and Astronomy, Ghent University, Proeftuinstraat 86/N12, B-9000 Gent, Belgium; 3 University of Manchester, Manchester, M1 7HS UK; 4 Northwestern University, Feinberg School of Medicine, Chicago, Illinois 60611 USA

**Keywords:** Imaging techniques, Physics, X-rays

## Abstract

We present a new method of single acquisition spectroscopic imaging with high spatial resolution. The technique is based on the combination of polychromatic synchrotron radiation and ptychographic imaging with a recently developed energy discriminating detector. We demonstrate the feasibility with a Ni-Cu test sample recorded at I13-1 of the Diamond Light Source, UK. The two elements can be clearly distinguished and the Ni absorption edge is identified. The results prove the feasibility of obtaining high-resolution structural and chemical images within a single acquisition using a polychromatic X-ray beam. The capability of resolving the absorption edge applies to a wide range of research areas, such as magnetic domains imaging and element specific investigations in biological, materials, and earth sciences. The method utilises the full available radiation spectrum and is therefore well suited for broadband radiation sources.

## Introduction

X-ray ptychography is a scanning coherent diffractive imaging (CDI) technique. The method produces quantitative phase images (electron density maps) with nanometre resolutions across extended fields of view^1^. The sample is scanned relative to the beam on a two-dimensional grid. The width of the beam and the grid spacing are selected such that neighbouring measurements overlap, in accordance with the relevant sampling condition^2^. At each scan point the far field scatter pattern is recorded on a two-dimensional array detector. In hard X-ray ptychography, single photon counting detectors (PCDs) are widely used for their high sensitivity and noise free detection capabilities^3–9^.

A conventional ptychographic acquisition requires a monochromatic beam of radiation. The addition of chemical and magnetic sensitivity, through the combination of ptychography with spectroscopy, has previously been demonstrated in both soft^10,11^ and hard X-rays^12–14^. These rely on the recording of several independent ptychographic datasets, as the monochromatic beam is scanned across the absorption edge of an element of interest.

PCDs have the ability to set energy thresholds with typical resolutions in the order of kilo electron-volts^15^. Their energy discriminating capabilities have been applied to real space spectroscopic imaging, including medical CT applications^16–18^. With ptychography this ability has been used to eliminate noise^19,20^. Recently developed hyperspectral detectors have the ability to detect every single incident X-ray photon with an energy resolution of hundreds of electron-volts. These systems too have been applied to real space spectroscopic imaging and, in a few cases, used to combine structural and chemical information^21–23^.

The proposed hyperspectral ptychographic imaging (HPI) method combines for the first time ptychography with broadband radiation and a hyperspectral detector. The hyperspectral detector replaces the energy selectivity of the monochromator, so that the full spectroscopic ptychography dataset can be recorded in a single acquisition.

## Methods

### Experimental setup

The experiment was carried out at the Diamond I13 beamline for imaging and coherence. The beamline’s emphasis is on multi-scale and multi-modal imaging for studies on the micro- and nano-lengthscale^24–26^. The beamline consists of two independently operating branchlines, here the I13-1 coherence branchline was used. I13-1 is dedicated to advanced coherent imaging methods, namely ptychography.

The front end of I13-1 is equipped with a 2.8 m long, 25 mm period undulator, a filter box, a three-stripe X-ray mirror (Pt-Si-Rh), and a Si111 double crystal monochromator (bandwidth of 10^−4^). The I13 long straight section has been modified to the so-called mini-beta^26^, allowing for an undulator gap down to 6.15 mm. The ptychographic end station is positioned in the experimental hutch at 220 m from the undulator X-ray source. The large distance between source and experiment provides a very large transverse coherence length^27^, which is tuneable through a set of source defining slits, positioned at a virtual source plane.

The sample stage consists of a 6-axis base motor on top of which sits a 3-axis piezo motor (P-563). The piezo motors are used for the ptychographic scanning and are operated in a virtual coordinate system, where the sample can be scanned in any plane.

The X-ray probe at I13-1 is formed through the Fresnel zone plate (FZP) focusing optics, (400 μm diameter with 150 nm outer zone width) in combination with a 60 μm diameter gold central stop (CS) and a 20 µm order sorting aperture (OSA). The CS is positioned immediately upstream of the FZP, with the OSA close to the focus. The spacious experimental hutch allows for a sample-detector distance of up to 14.5 m. Beam widths in the range of 200 nm–20 μm are typically used, facilitating a wide variety of experimental geometries. For this experiment, the hyperspectral SLcam^28,29^ was placed 4.05 m downstream of the sample and the beam width was between 11 and 2 µm across an energy range of 8227 to 8461 eV.

The experiment began with a fine tuning of the energy calibration of the SLcam using a monochromatic beam of 8339 eV (Ni K-edge), with the Si strip of the mirror attenuating the higher order undulator harmonics. The bandwidth of the detector was measured to be 172 eV (FWHM) by deconvoluting the recorded spectrum by the known bandwidth of the monochromator. Additionally, a baseline ptychographic measurement was performed at this energy.

As the proposed HPI method makes use of a broadband X-ray beam, the monochromator was removed after these preliminary monochromatic measurements. The resulting polychromatic X-ray beam is characterized by several undulator harmonics. The undulator was tuned to have the third harmonic centred on the Ni K-edge. The other undulator harmonics were attenuated through a combination of the Si mirror strip and a set of filters: 14 μm Cu, 1.34 mm pyrolytic graphite, and 280 μm Al.

**Figure 1:**
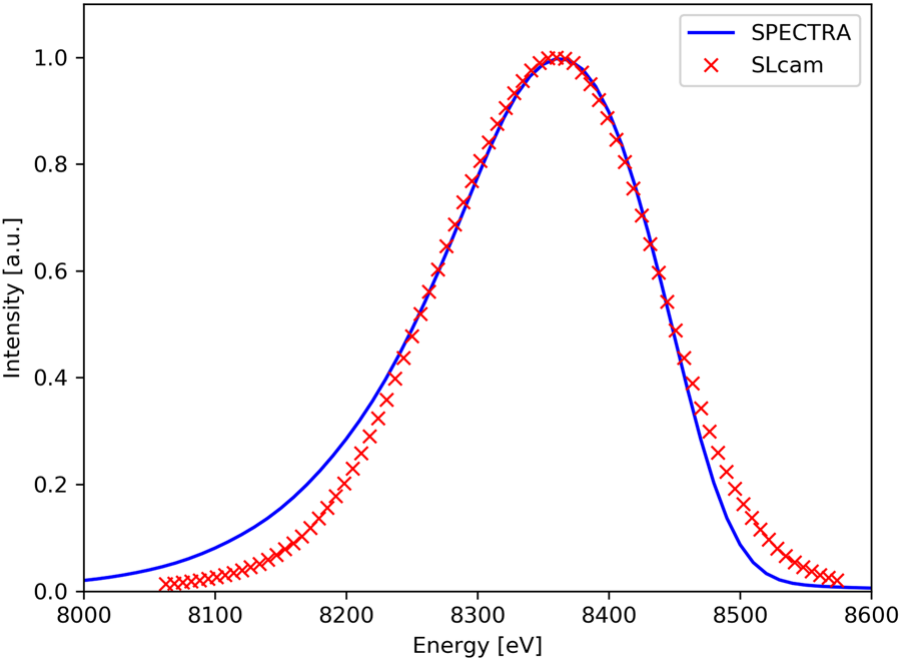
Measured and simulated off-axis X-ray spectrum. The red crosses represent the measured data taken by the SLcam, after a deconvolution of its spectral response. The simulation (blue line) was created using SPECTRA code^30^ with 25 µrad offsets in the horizontal and vertical directions.

The beamline offers extensive possibilities to explore the parameter space for new experimental methods. Here, the energy bandwidth of the radiation was increased further by offsetting the X-ray beam relative to the optical axis of the beamline by 25 µrad, both horizontally and vertically. Simulations and measurements of the resulting harmonic spectrum around the Ni K-edge is shown in Fig. 1. Both simulations and measurements indicate a bandwidth of 180 eV. The measurement is taken from the deconvoluted spectra of the SLcam and the simulations were carried out with the SPECTRA code^30^.

### Hyperspectral detector

The SLcam is based on a pnCCD with a fully depleted 450 μm thick silicon active volume. The detector contains 528 by 264 pixels of 48 μm pixel pitch, of which only a central area of 264 × 264 pixels is illuminated. The signal from the illuminated area is transferred to the shielded area in 25µs, from where the signal is read out at a frame rate of approximately 400 Hz. The sensor is coupled to 11 bit ADCs through front-end electronics with low noise characteristics, enabling the detector to reach an energy resolution of approximately 150 eV at the Mn Kα peak. This performance is comparable to state-of-the-art single-pixel energy dispersive detectors.

The incident X-ray photon generates an electrical charge on the central pixel and also on neighbouring pixels. The position of the photon is determined with sub-pixel accuracy from the centre of mass of these charge clusters, its energy by the total deposited charge on the detector^31^. It is noteworthy that the accuracy of the position of the photon is better than the pixel size, and images can be produced at variable pixel sizes^29,32^.

The raw data is read out using an in-house developed acquisition framework^33^, which contains an acquisition chain that transforms raw detector frames into a stream of photon events. All information contained in these events is floating point based, allowing for arbitrary binning both in spatial and energy dimensions. This flexible rebinning is what allows for easy implementation of arbitrary pixel grids^34^ (usually an even multiple of the physical grid), and provides the means to tailor the energy bins to the specific requirements of an experiment. The software suite provides further support for storing raw unprocessed detector data, allowing for later reprocessing in the case of improved algorithms, detector calibration, or new post-processing steps. Finally, a comprehensive control GUI is provided, along with a set of diagnostic utilities to verify detector and experimental setup performances.

In order to avoid the superposition of charge clusters generated by the incoming photons, the photon flux on the detector should be below 5 photons pixel^−1^ s^−1^ for a broadband (white) spectrum^22^. For small bandwidth spectra (either quasi-monochromatic or spiked), this count rate can be increased as the pileup signal is less complex (i.e. pileup only gives a signal at energies above the spectral region of interest). For this experiment, the photon flux on the detector was kept below 10% pixel fill rate (corresponding to 10 photons pixel^−1^ s^−1^) in the brightest regions by tuning the width of the front end slits.

### Fluorescence mapping

Beside the far-field diffraction patterns, the X-ray fluorescence spectra emitted from the sample at each scan position was recorded for validation of the proposed HPI method. The fluorescence detector, a single element Vortex®-60EX silicon drift X-ray detector, was placed in the sample plane, perpendicular to the beam direction. A fluorescence map of an element of interest is generated by selecting the relevant fluorescence emission line.

### Data processing

I13-1 has online ptychography reconstruction capabilities built on *PtyREX*, the reconstruction package for electrons and X-rays^35^, which is used for the processing and analysis of the ptychographic data. The hyperspectral data from the SLcam were processed after splitting the spectrum into 26 eV wide channels, chosen as a compromise between spectral resolution and photon statistics. A sampling step smaller than the detector resolution is possible because the accuracy of the energy centroid is much greater than the energy resolution of the detector^33^. This is confirmed by the capability of reproducing the energy resolution by convolution (see Figs 1 and 3b). Each energy channel from the detector was processed individually through 100 iterations of ePIE^36^, with position correction^37^ and, where required, up-sampling^38^.

**Figure 2:**
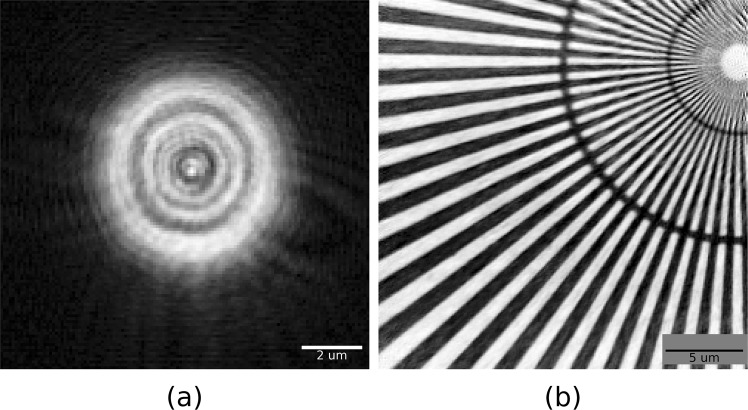
Reconstruction of ptychographic data, recorded with monochromatic beam (8339 eV) and the SLcam hyperspectral detector. (**a**) Probe modulus. (**b**) Object phase. The object is a Siemens star test sample, with radial markers representing 500, 200, and 100 nm line spacings. The reconstructed pixel size is 96 nm, the field of view is 24 × 24 µm^2^, and the resolution is sub 200 nm.

**Figure 3:**
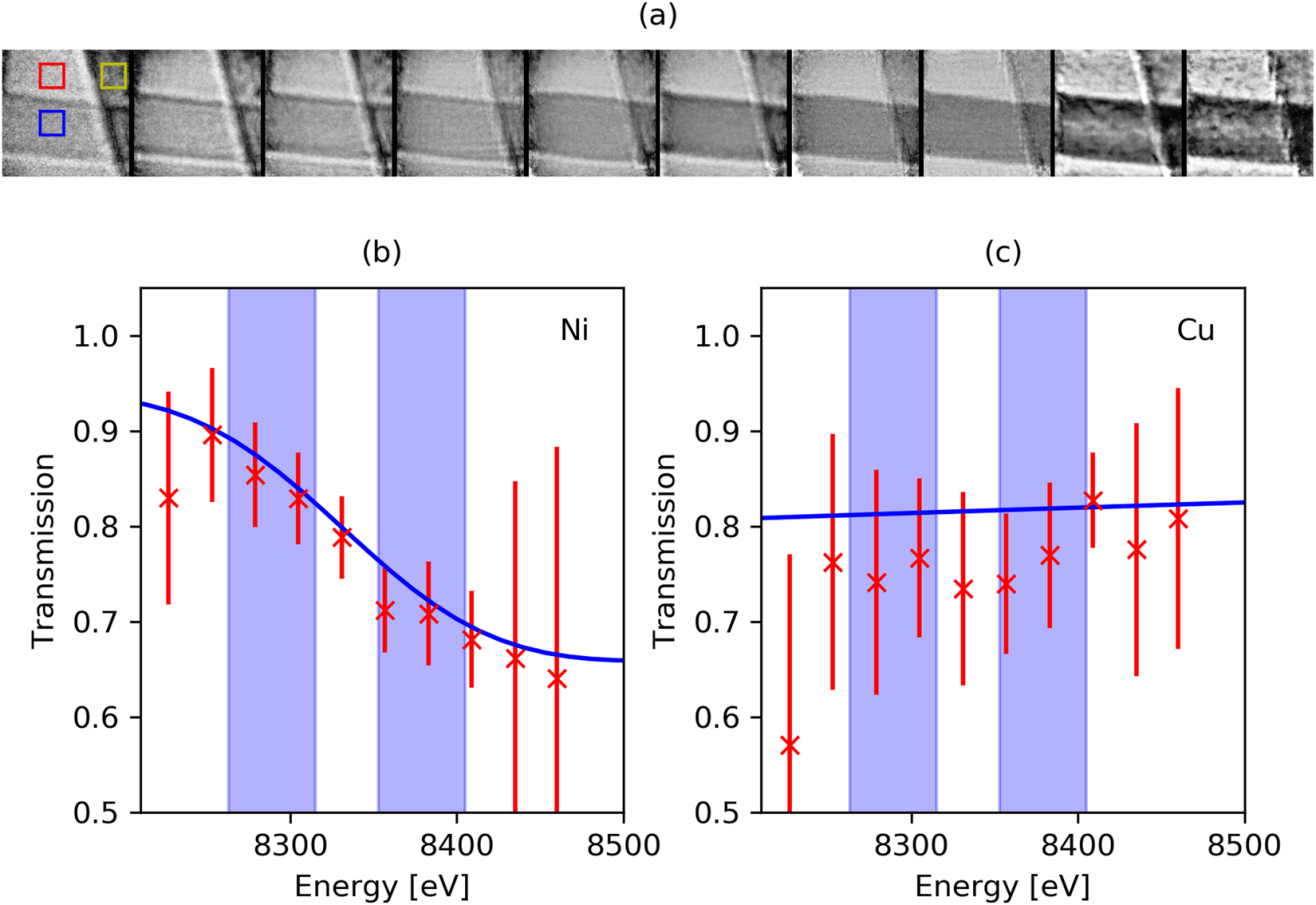
(**a**) Normalised transmission images from the hyperspectral ptychographic reconstruction. From left to right the energy is increasing from 8227 to 8461 eV in 26 eV steps. The red crosses show energy-transmission profiles through 1.5 µm of Ni (**b**) and 5 µm of Cu (**c**). The blue lines in (**b**,**c**) show the theoretical values from Henke^48^ convoluted with the detector response and energy binning. The blue vertical bands mark the energy bins used later for the edge subtraction.

The initial beam profile was modelled for each energy separately, given the chromatic nature of the optics used. The beam size varies within the studied energy range (8227 to 8461 eV) between 11 and 2 µm, which has an effect on both the real space overlap and the reciprocal-space sampling condition. These variations have a significant impact on the quality of the images, particularly where the deviation from the nominal 6 µm beam is greatest. Care must be taken to account for these effects during both the data acquisition and reconstruction processes. Where the reciprocal-space sampling condition was not fully satisfied, because the beam was too large^2^, up-sampling^38^ was used in the reconstruction process. For the cases where the beam size was too small, compared to the step size, the data has been neglected.

The result of the data post processing is a complete hyperspectral ptychography reconstruction, including the complex beam profile and sample image for each energy channel.

## Results

We describe here the first results of single acquisition hyperspectral ptychographic imaging. The SLcam was initially characterised for ptychographic imaging with monochromatic radiation. The beam was condensed to a spot size of 6 μm. The Siemens star test sample was scanned on a 16 × 16 raster grid with a step size of 1.5 μm and a detector exposure time of 80 s. The reconstructed probe modulus and object phase are shown in Fig. 2. The experimental geometry gives a pixel size in real space of 96 nm and the resulting image resolution is sub 200 nm. The following spectroscopic HPI measurements were recorded with the polychromatic beam, with a spectrum as shown in Fig. 1.

### Edge identification

To evaluate the feasibility of the HPI method, a Cu-Ni grid pair with bar widths of 12.5 µm was used. The thicknesses of the grids were 5 µm and 1.5 μm, respectively. The ptychography scanning parameters used for the Cu-Ni grid were the same as for the Siemens star. The hyperspectral ptychographic image was used to produce an energy-transmission plot of the Ni and Cu around the Ni absorption edge at a spectral bin-width of 26 eV. The modulus of the reconstructed objects and the corresponding energy-transmission plot for the two elements are shown in Fig. 3. The transmission has been evaluated by averaging the intensity over an area of 50 × 50 pixels in the air, Ni, and Cu (red, blue and yellow boxes in Fig. 3a). The error bars are calculated from the standard deviation of the average. The large error bars at low and high energy are due to poor photon statistics at the edges of the undulator harmonic. The energy-transmission plot shows that the Ni absorption edge is clearly identified.

### Edge subtraction

K-edge subtraction is a method of discriminating between elements within a sample^39^. The method takes two images of the same sample region, recorded with illumination energies above and below the absorption edge of an element of interest. The difference of these images produces a map that represents the distribution of the element across the sample.

**Figure 4:**
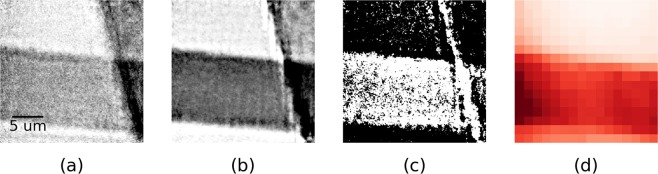
Single acquisition elemental mapping of hyperspectral ptychographic data. Ptychographic transmission images below (**a**) and above (**b**) the Ni K-edge (8289 eV and 8379 eV, respectively). (**c**) K-edge subtraction of (**a**,**b**), showing the distribution of Ni. (**d**) The fluorescence signal from Ni (7478 eV), confirming the horizontal orientation of the Ni grid in (**c**).

As shown in Fig. 3, the hyperspectral ptychography reconstruction contains several images both above and below the absorption edge of the Ni. Two particular images, corresponding to central energies of 8289 eV and 8379 eV (indicated by blue vertical bands in Fig. 3b,c) were selected in order to demonstrate the principle on our single acquisition dataset. The width of the channel above and below the edge was widened from 26 to 52 eV to improve the photon statistics. It is worth noting that this rebinning is achieved purely from the reprocessing of the SLcam’s raw data and no rescanning is required. The result of the K-edge subtraction on the ptychographic transmission images is shown in Fig. 4c. The resultant HPI image clearly shows that the horizontal bar is Ni. However, some edge artefacts exist, generating a false Ni signal across the edge of the vertical Cu grid bar. The edge artefacts appear more severe at the higher energies, where the effects of the reduced beam size are most prominent. This can be overcome with achromatic optics (not available during this experiment).

As well as the spectro-ptychographic data, a fluorescence image of the Ni-Cu grid was recorded, using the same scanning parameters but with 1 s exposure. The result is a Ni map confirming the elemental distribution as shown in Fig. 4d, at a resolution limited by the spot size of the irradiating beam. Ptychographic images are freed from this constraint and are instead limited by the effective numerical aperture of the detector and the stability of the instrumentation. The proposed method computes the elemental maps directly from the ptychographic images, on a pixel by pixel basis, and therefore capable of the same high-resolutions.

## Discussion

The reported experiment is the first case of a hyperspectral detector being combined with ptychographic imaging methods and a broadband spectrum. Initially, a monochromatic beam and a Siemens star test sample were used to characterise both the detector’s spectral resolution, which was measured to be 172 eV, and ptychographic spatial resolution, which was measured to be sub 200 nm. The experiment was then switched to polychromatic beam for the spectroscopic measurements. A Cu-Ni grid was chosen as a test sample for the HPI measurements. We succeeded in identifying the Ni K-edge and discriminating between the Ni and Cu via K-edge subtraction using the transmission maps.

Unlike other methods such as fluorescence imaging with nano-probes^40,41^, or spectral ptychography through energy scans^10–14^, the proposed technique gives access to spectral information at resolutions beyond the beam size from a single two dimensional scan. The focus of this study was on the spectral capabilities of the technique and the chosen sample is not sufficient for a meaningful evaluation of the spatial resolution. However, there exists a relationship between spatial resolution and bandwidth^42^, which suggests a limitation here below 200 nm. We intend to investigate the topic further in future experiments.

The experimental method will be optimised with further adaptations to the instrumentation. An achromatic optic, such as a KB-mirror system will provide a constant spot size over the entire energy range, improving the data quality across the spectrum. The method makes use of broadband radiation, which could be achieved more effectively by tapering the undulator, flattening the spectral distribution of the harmonic^43^. These combined improvements would increase the overall image quality and energy resolution of the technique, potentially expanding the method to applications that require a higher sensitivity, such as L-edge subtraction and magnetic domain imaging.

The nature of the SLcam currently limits the total count rate to around 10 photons pixel^−1^ s^−1^, which resulted here in an acquisition rate (for the whole spectral dataset) of 35.5 s µm^−2^. This matches the acquisition rate recently reported for an energy scanning experiment using a conventional PCD^12^. The total count rate of the experiment would be increased by using either a diffuser (spreading the total number of recorded photons across many more pixels) or with upcoming faster hyperspectral detectors with improved radiation hardness^44^. As all the energy channels are recorded simultaneously, the effect of any optical or sample drift occurring in between scans is removed, simplifying the post processing procedures. The method could also be extended to multiple energy threshold detectors^45^, that already exist with acquisition rates up to 30 Hz^46^, albeit at lower energy resolutions. In that case, multiple elements could be targeted depending on the number of thresholds available and on the energy resolution of the detector.

In summary, the HPI method provides elemental maps directly from high-resolution ptychographic data, without the need for high quality monochromating systems, high quality focusing optics, or repeated sample scanning. The SLcam is well suited for measurements with low brilliance. The brilliance required for the reported experiment is 3 × 10^11^ photons s^−1^ mm^−2^ mrad^−2^ 0.1%BW which is comparable with the brilliance of liquid metal-jet based x-ray sources^47^ making the technique applicable to laboratory sources. Many applications would benefit in areas such as magnetism, biology, earth and materials sciences.

## Data Availability

The authors confirm that all of the data used in this study are available without restriction. Data can be obtained by contacting darren.batey@diamond.ac.uk.
